# Supplementary material for: A UMLS-based spell checker for natural language processing in vaccine safety
Source: BMC Med Inform Decis Mak. 2007 Feb 12;7:3. doi: 10.1186/1472-6947-7-3 (PMC1805499; doi:10.1186/1472-6947-7-3)
Supplement: Additional file 13 — RAP application directory. Sets up directory for RAP (RDF application for PHP) source code [file 1472-6947-7-3-S13.gz › rap/doc/tutorial/visualization_tutorial.htm]

Graph Visualization


### RDF API for PHP V0.9.2

# Tutorial: Graph Visualization

This turorial is part of the RAP - Rdf API for PHP documentation.

  

Anton Köstlbacher <ak@semaweb.org>
  
April 2005

This is a short Tutorial on how to use GraphViz *dot* within RAP to
easily generate visualizations of models in several file formats like png, svg or jpeg.

### Introduction

The visualization tool for RAP V0.9.2 mainly consists of the method `RDFUtil::visualizeGraph()`. It prepares a so called dot-file, which is afterwards processed by the *dot*-application.
GraphViz *dot* is freely available under the Common Public License. The (binary or ascii) output of the *dot*-application is directly passed back to the browser. A working demo can be found in the RAP distribution (/rdfapi-php/test/custom\_test\_viz.php).

### Installation

Before we get started we need to download and install GraphViz *dot* into a directory on the machine, on which RAP is running. GraphViz *dot* is available as source code and binaries for several operating systems at http://www.graphviz.org. Additionally we need to set up a directory for temporary files which is read- and writeable by the webserver-software. Take care of security issues, if you are using RAP on a publicly accessible machine!

### Configuration

At first we have to make sure, that the webserver (which runs RAP) can access the
GraphViz *dot*-binary, otherwise we can
only generate dot-files and use them for manual input to the Graphviz standalone application.
For immidiate generation of images we need to set the following parameters in

**constants.php**

```` ```
// ----------------------------------------------------------------------------------
// GRAPHVIZ
// ----------------------------------------------------------------------------------

// path to the dot binary
define('GRAPHVIZ_PATH', 'C:\/Programme\/ATT\/Graphviz\/bin\/dot.exe');

// directory for temporary files
// Attention: must be write-/readable by the webserver
define('GRAPHVIZ_TEMP', 'C:\/');

// display statistical data in generated images
// currently only number of statements drawn
define('GRAPHVIZ_STAT', TRUE);

// allowed file formats
// for security reasons (to prevent code injection)
define('GRAPHVIZ_FORMAT', 'svg, dot, jpg, png, gif, vrml');

// enable clickable URIs
// only supported by certain formats (e.g. SVG)
define('GRAPHVIZ_URI', FALSE);

// define parameters for the graphical output
// if a paramter is undefined, the default value of graphviz is used
// for further information see: http://www.graphviz.org/Documentation.php
$graphviz_param = array(
   GRAPH_STYLE     => 'rankdir="LR"',
   RESOURCE_STYLE  => 'style="filled",color="#FFD800",fontname="Courier",fontsize="10"',
   LITERAL_STYLE   => 'shape="box",style="filled",color="#B7FFAF",fontname="Courier",fontsize="10"',
   PREDICATE_STYLE => 'fontname="Courier",fontsize="10"',
   INFERRED_STYLE  => 'style="dotted",fontname="Courier",fontsize="10"',
   BLANKNODE_STYLE => 'style="filled",color="#DDDDDD",fontname="Courier",fontsize="10"',
   BOX_STYLE       => 'fontname="Courier",fontsize="8",color="#BBBBBB"'
);
``` ````

Here you may find detailed explanations about the STYLE-paramters.
Or you simply try to carefully change the above given values.  
The supported file formats can be found here.

### Visualizing Models

Now we are keen on generating our first graph visualization and here we go:

```` ```
// Include RAP
define("RDFAPI_INCLUDE_DIR", "./../api/");
include(RDFAPI_INCLUDE_DIR . "RDFAPI.php");

// Filename of an RDF document
$base="example1.rdf";

// Create a new MemModel
$model = ModelFactory::getDefaultModel();

// Load and parse document
$model->load($base);

// Visualize model
header('Content-type: image/gif');
$model->visualize('gif');
``` ````

As you see above, there is an alias for `RDFUtil::visualizeGraph()` in the model-class, which makes usage really convenient. Note that the code above will instantly return binary data, so we have to set the correct header for the output file: `header('Content-type: image/gif');`. If you choose to generate, for example svg, which is a xml-based format, you would need to set the header to `header('Content-type: image/svg+xml');`.  
Note: Do not send any headers before. Headers are sent automatically, if there are for example whitespaces before: `<?php`. To prevent problems with headers, turn on output\_buffering in your php.ini or use output-buffering manually.

This is in fact almost everything you need to know to visualize a model. But we should have a short look at the parameters we can pass to the method:

```` ```
// Visualize model
$model->visualize('gif', FALSE);
``` ````

Adding FALSE as second argument will disable the detection of Namespaces and you get fully qualified URIs as you see below:

### Performance and Alternative Visualization Engines

The implemented Method in RAP itself can handle very large Models, but visualizing large and/or complex models can take a few minutes, because of the dot-application. The duration depends, as you might assume, on factors like the power of your machine, the size and/or complexity of the model and the chosen output format. I recommend to use XML-based formats like SVG for large models and set an additional header, that forces a 'save as...' window:

```` ```
// Visualize model
header('Content-type: image/gif');
header('Content-Disposition: attachment; filename=example1.gif');
$model->visualize('gif');
``` ````

You can also get the original dot-file and pass it for example to the gui-version of Graphviz:

```` ```
// Visualize model
header('Content-type: text');
$model->visualize('input_dot');
``` ````

In future versions there will eventually be integrated support for alternative visualization engines like Tulip. At present you can use the dot-file and import it manually to your preferred visualizer. Most of the visualizers support the dot-format. See: http://directory.google.com/Top/Science/Math/Combinatorics/Software/Graph\_Drawing/
